# Horseback Riding Improves the Ability to Cause the Appropriate Action (Go Reaction) and the Appropriate Self-control (No-Go Reaction) in Children

**DOI:** 10.3389/fpubh.2017.00008

**Published:** 2017-02-06

**Authors:** Nobuyo Ohtani, Kenji Kitagawa, Kinuyo Mikami, Kasumi Kitawaki, Junko Akiyama, Maho Fuchikami, Hidehiko Uchiyama, Mitsuaki Ohta

**Affiliations:** ^1^Laboratory of Effective Animals for Human Health, Azabu University School of Veterinary Medicine, Chuo-ku, Kanagawa, Japan; ^2^Faculty of Animal Health Technology, Yamazaki Gakuen University, Hachioji, Tokyo, Japan; ^3^Laboratory of Animal Facilitated Therapy, Faculty of Agriculture, Tokyo University of Agriculture, Atsugi, Kanagawa, Japan

**Keywords:** horse riding, Go/No-go tasks, arithmetic problems, heart rate, autonomic nervous activity, three-dimensional acceleration

## Abstract

**Background:**

There are many obvious health benefits to riding, including developing a strong core and legs, but there are also many less obvious benefits, such as increased confidence and introspection. Few studies have addressed the effects of horseback riding on children and the mechanisms underlying how riding affects humans. We examined the effects of horseback riding on the ability to distinguish Go/No-go tasks and solve arithmetic problems in children.

**Methods:**

The subjects were 34 boys and 72 girls, aged 10–12 years old, which were divided into three groups (horse riding, walking, and resting). They were healthy typical children, who performed the Go/No-go tasks and solved the arithmetic problems. The heart rate and heart rate variability of the children, and the three-dimensional acceleration of the children while walking horses, were examined.

**Results:**

Riding on a half-breed horse or a pony improved the ability to perform Go/No-go tasks and solve arithmetic problems, possibly through sympathetic activity. Some horses, like the Kiso, might provide a healing effect to children through parasympathetic activity. Statistically significant differences in the three-dimensional acceleration and the autonomic activities were observed among the three horses. The acceleration in the Kiso horse group during walking in hand was significantly different from those involving the other two horses, indicating that the vibrations produced by these horses might modify the autonomic activities.

**Conclusion:**

The most important beneficial factor of horseback riding for children and for human health appears to be associated with the horse’s vibrations, which may differ among horses. Riding some horses may improve the ability of children to respond with an appropriate action depending on the situation (Go reaction) or use self-control appropriately (No-go reaction), possibly through the activation of the sympathetic nervous system.

## Introduction

Equine-assisted interventions are used in many institutions worldwide for the treatment of individuals with mental and physical disabilities (1, 2), as well as to improve the health of all people (3–5). Many reports have demonstrated the benefits of horseback riding with respect to enhancing overall health, improving circulatory functions (6), providing stimulation of the spinal cord, promoting the development of balance and motor functions (7), and improving muscle strength (8, 9). Moreover, horseback riding has mental effects, such as relieving anxiety (10), reducing hyperactivity (11), enhancing self-esteem (12), and promoting relaxation (13). However, few studies have addressed the effects of horseback riding on children and the mechanisms underlying how the riding affects humans. To increase the popularity of horseback riding, it is necessary to clarify how it benefits the riders. The movement of the horse’s pelvis may provide motor and sensory inputs to the human body. The reciprocal movements of a walking horse produce a pelvic movement in the rider’s body that closely resembles human ambulation (14–16).

There are four types of horse steps: the walk, trot, canter, and gallop (17). The walk is the slowest step, landing the left hind leg, left foreleg, right hind leg, and right foreleg in order, creating a four-beat rhythm. The trot involves two beats, two limbs in a diagonal sequence landing at the same time and occurs at a higher speed than the walk. These steps (the walk and the trot) are mainly used in equine-assisted interventions. One important characteristic of the horse steps is that they produce three-dimensional accelerations (16).

In this study, we examined the effects of horseback riding on the performance of Go/No-go tasks and arithmetic problems in children and measured the three-dimensional acceleration in three different kinds of horses with riders. The Go/No-go task is a behavioral test that determines the ability to take appropriate action (Go reaction) and to show appropriate restrain (No-go reaction) depending on the situation. Additionally, we analyzed the relationship between the accelerations and a physiological index. A power spectrum analysis of heart rate variability (HRV) provides a quantitative non-invasive means of assessing the function of the cardiovascular system in the short term, representing the beat-to-beat variation in the heart rate generated by the interplay of sympathetic and parasympathetic nerve activities (18). The sympathetic response is fairly sudden in order to prepare the body to respond to an emergency situation or acute stress, short term stressors (19). The spectral analysis of HRV is divided into two major oscillatory components; the high-frequency (HF) domain (0.15–1.00 Hz), which reflects parasympathetic activity, and the low-frequency (LF) domain (0.04–0.15 Hz), which reflects both sympathetic and parasympathetic activities. To elucidate the mechanism underlying the beneficial effects of horseback riding on children, we examined the autonomic activities of riders using HRV.

## Materials and Methods

### Ethics

All of the experiments in this study received written informed consent for children and their parents and were approved by the Human Research Ethics Committee (Approval number #2738) and Animal Experiment Ethics Committee (#080618-1) of Azabu University in accordance with the World Medical Association Declaration of Helsinki.

### Subjects

In total, 106 children (34 boys and 72 girls, 10–12 years old), consisting of 80 students from an elementary school’s upper grades (fourth, fifth, and sixth graders) and 26 primary school students (fourth, fifth, and sixth graders) in Sagamihara City, were gathered by public recruitment. They had no history of neurological or psychological impairment. They heard an overview of the experiments and then decided to volunteer. They gave informed consent and were comfortable in having limited riding experience. Of those, 54 children (15 boys and 39 girls), who had ≤1 horse-riding experience, were the subjects for the horse-riding experiments. They were separated into three subgroups, A, B, and C horses. There were 5 boy and 13 girl riders in each subgroup. The other students (19 boys and 33 girls) were used in the walking (9 boys and 17 girls) and the resting (10 boys and 16 girls) groups.

A Polar^®^ RS800CX digital HR monitor and a Polar^®^ Wearlink strap with a transmitter (Polar^®^ Electro Öy, Kempele, Finland) were attached to the left wrist and the chest, respectively, of each subject to measure the HR and HRV. The sensors (4.5 cm × 4.5 cm × 1.2 cm; MVP-RF8-GC-2000; Microstone, Nagano, Japan) for monitoring accelerations were attached to the backsides of the children. Half of the children in each group, 13 each in the resting and walking groups, and 9 each in the riding groups, were randomly chosen for the attachment of the HR monitor and transmitter. However, some of the children had difficulty attaching those instruments and thus were replaced with the experiment on monitoring accelerations.

### Horses

We used three different kinds of horses, with a mean age of 20 years (range 18–22) in this study: a half-breed horse (mare) that was 155-cm high and 190-cm wide (horse A); a Kiso (gelding), which is a Japanese traditional horse, that was 141-cm high and 184-cm wide (horse B); and a pony (gelding) that was 135-cm high and 171-cm wide (horse C). The horses were kept in individual stalls (4 m × 4 m) with natural lighting at Azabu University (Kanagawa, Japan) and trained to be ridden by the disabled and children mainly by two of us, who had over 10 years experience with horse training. The horses were also ridden almost every day by Azabu University students. The horses had no history of medical problems in the preceding 6 months, had not received any pharmacological treatment for 3 months prior to the study, and were considered to be clinically healthy by the two veterinarians.

### Experimental Procedures

Horseback riding and walking, as the control, was carried out at the 50 m × 50 m outdoor field at Azabu University.

**Figure 1:**
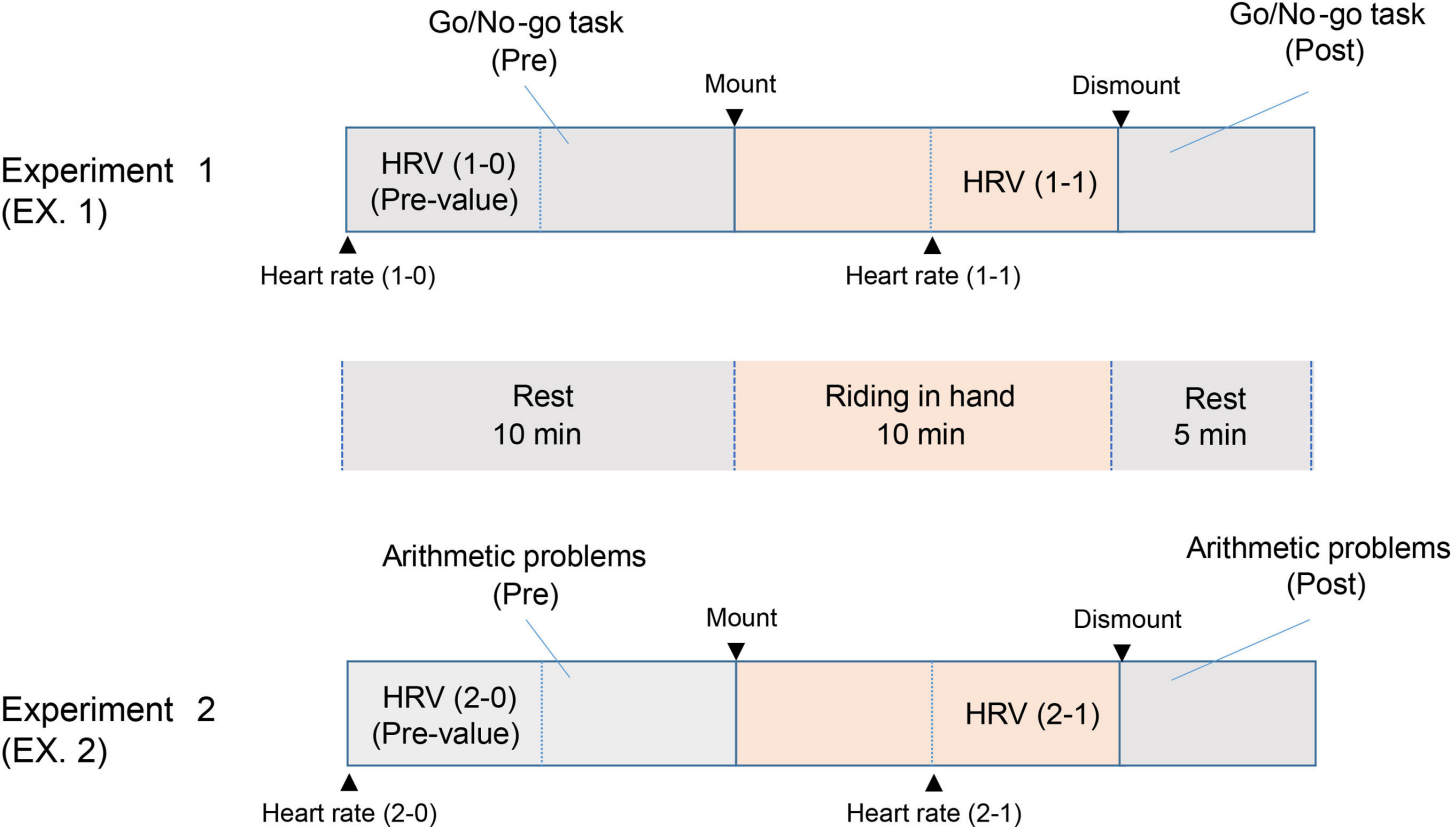
**Diagrams of experimental procedures showing 10 min rest, 10 min first treatment, the Go/No-go tasks (Experiment 1), 10 min rest, 10 min second treatment, and the arithmetic problems (Experiment 2)**. The heart rate variability (HRV) measurements were taken during the rest [pre-values: HRV (1-0) and HRV (2-0)], the first treatment [HRV (1-1)] period (EX. 1), and the second treatment [HRV (2-1)] period (EX. 2). The Go/No-go tasks were performed twice: during the 10 min rest (Pre) and immediately after the first 10 min of riding, walking, or resting (Post, EX. 1). The arithmetic problems were also solved twice: during the 10 min rest (Pre) and immediately after the second 10 min session (Post, EX. 2).

All of the subjects rested on the seats along the field’s fence for 10 min and performed the Go/No-go pre-task (Pre) during the rest (Figure 1). The riding, or the walking, was performed for 10 min with a 5 min rest to perform the Go/No-go post-task (Post). The tasks were applied to all of the subjects, including the resting group, and performed just after the 10 min of riding, walking, or resting (Experiment 1). After the task, the riding and the walking were resumed, along with a 10 min rest (Figure 1). All of the children completed the arithmetic problems after the second 10 min session of riding, walking, or resting (Experiment 2).

### Experiment 1 (EX. 1): Go/No-Go Tasks

The tasks were presented in three colors, red, blue, and yellow, serially on a 15″ computer screen (Sony, Tokyo, Japan) for 200 ms each. The “Go” reaction required the pressing of the space bar on the keyboard when blue or yellow was displayed, while the ‘No-go’ reaction required that no key was pressed for more than 2,000 ms when red was displayed. Incorrect answers were recorded if the “Go” reaction/color was displayed and the space bar was not pressed for more than 2,000 ms, and if the “No-go” reaction/color was displayed and any key was pressed. The colors were presented, in a random order, 10 times each. In addition, the intervals between the color presentations were chosen at random from 2,000, 2,500, or 3,000 ms. In this experiment, the children were seated on a chair placed in front of the 15″ monitor with the keyboard on a desk after the first 10 min of riding, walking, or resting (Figure 1).

### Experiment 2 (EX. 2): Arithmetic Problems

Thirty problems of 1-digit + 1-digit addition were randomly chosen and printed on one side of an A4-size paper (210 mm × 297 mm). The answers were written on the printed side of the paper. Children were seated after the second 10 min session of riding, walking, or resting and then given 30 questions to answer (Figure 1). They pressed the space bar of the computer just before answering and then pushed the bar again when they had finished the problems. The computer calculated the duration, in seconds, that the children spent on the arithmetic problems.

### Three-Dimensional Accelerations

The three-dimensional accelerations of the three horses were measured using an accelerometer. The sensors for monitoring accelerations were attached to the backsides of the children, and the data from the sensors were sent to a tablet computer (Acer Iconia TAB-W500S, New Taipei City, Taiwan) wirelessly through Bluetooth (Parani-UD100, Anaheim, CA, USA), over a distance of ~25 m. Nine of 18 riders per horse participated in the measurement of three-dimensional accelerations.

### Heart Rate Variability

The HR monitor and the strap with a transmitter (Polar^®^ Electro Öy, Kempele, Finland) were used to measure the inter-beat (RR) intervals. The data were filtered using Kubios HRV software, version 2.0 (The Biosignal Analysis and Medical Imaging Group at the Department of Physics, University of Kuopio, Kuopio, Finland), and the power spectrum for the frequency was calculated.

The measurement of the HRV was started just before the rest. The values were calculated from data captured from 5 min of each session, just before the task or the arithmetic problems [pre-values, HRVs (1-1) and (2-1), Figure 1].

### Statistics

Statistical analyses of the effects of horseback riding on the Go/No-go tasks and on the arithmetic problems, whose performance rates are shown as percentages with non-normal distributions, were performed using the appropriate Kruskal–Wallis, Mann–Whitney, or chi-squared test, and Welch’s test after a one-factor ANOVA. Spearman’s rank correlation coefficient between the Go/No-go tasks and the HRs was also calculated. Statistical analyses of the HRV data were carried out using the Wilcoxon signed-ranks test to compare values obtained during riding, walking, or resting with the pre-values. The significant differences between the accelerations were determined using Welch’s *t*-test. *P* values < 0.05 or better were considered significant.

## Results

The children were not injured by any factors, such as sensors or horseback riding, in this study.

### Go/No-Go Tasks and Arithmetic Problems: EXs. 1 and 2

**Figure 2:**
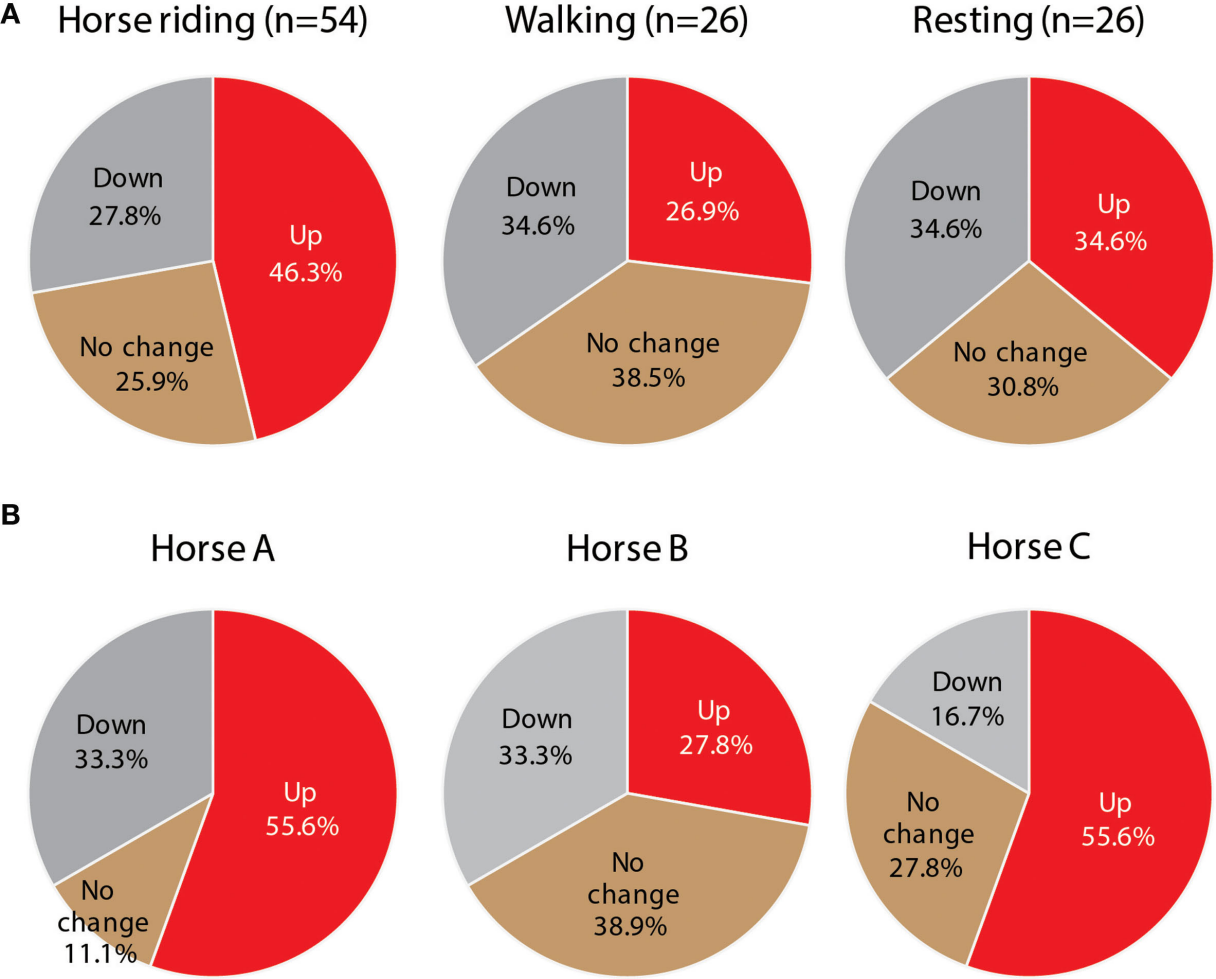
**(A)** The performance rate of Go/No-go tasks in children riding horses (*n* = 54), walking (*n* = 26), or resting (*n* = 26) is shown as percentages. “Up” indicates higher performance, and “Down” indicates lower performance. **(B)** The performances grouped by horse (A, B, and C) are shown as percentages (*n* = 18).

Horseback riding produced better Go/No-go results than walking or resting (Figure 2A). In 25 of 54 children (46.3%), the performance of Go/No-go tasks increased after 10 min of riding, while it improved in 7 of 26 children after 10 min of walking (26.9%). There were significant differences among the horses in the effects of riding on the Go/No-go tasks (Figure 2B). Riders of horses A and C performed better than those of horse B (A vs B, χ^2^(2) = 30.7, *P* < 0.01; B vs C, χ^2^(2) = 16.3, *P* < 0.01).

**Figure 3:**
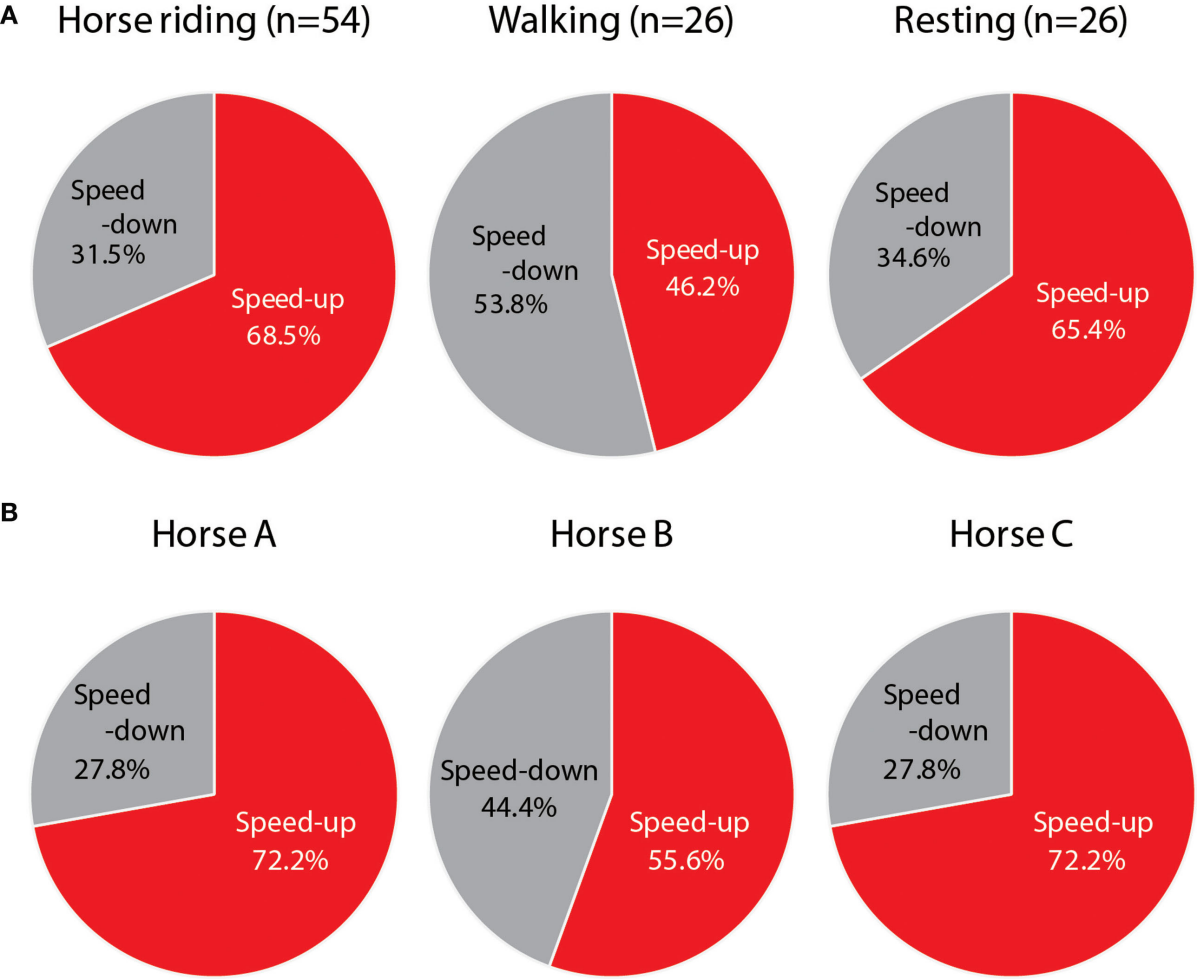
**(A)** The performance rate of arithmetic problems in children riding horses (*n* = 54), walking (*n* = 26), or resting (*n* = 26) is shown as percentages. “Speed-up” indicates greater achievement, and “speed-down” indicates lower achievement. **(B)** The performances grouped by horse (A, B, and C) are shown as percentages (*n* = 18).

In the pre-tasks, 17.9% of all subjects had incorrect answers for a single question and 2.8% of them had two incorrect answers, while in the post-tasks, 16.0% of all subjects had single incorrect answers and none of them had two (or more) incorrect answers. There was no significant difference in the solving of arithmetic problems between the pre- and the post-tasks (Figure 3A). However, riding on horses A and C shortened the testing time significantly (Figure 3B), compared with riding on horse B (A vs B, χ^2^(1) = 4.7, *P* < 0.05; B vs C, χ^2^(1) = 4.5, *P* < 0.05).

### HR

**Figure 4:**
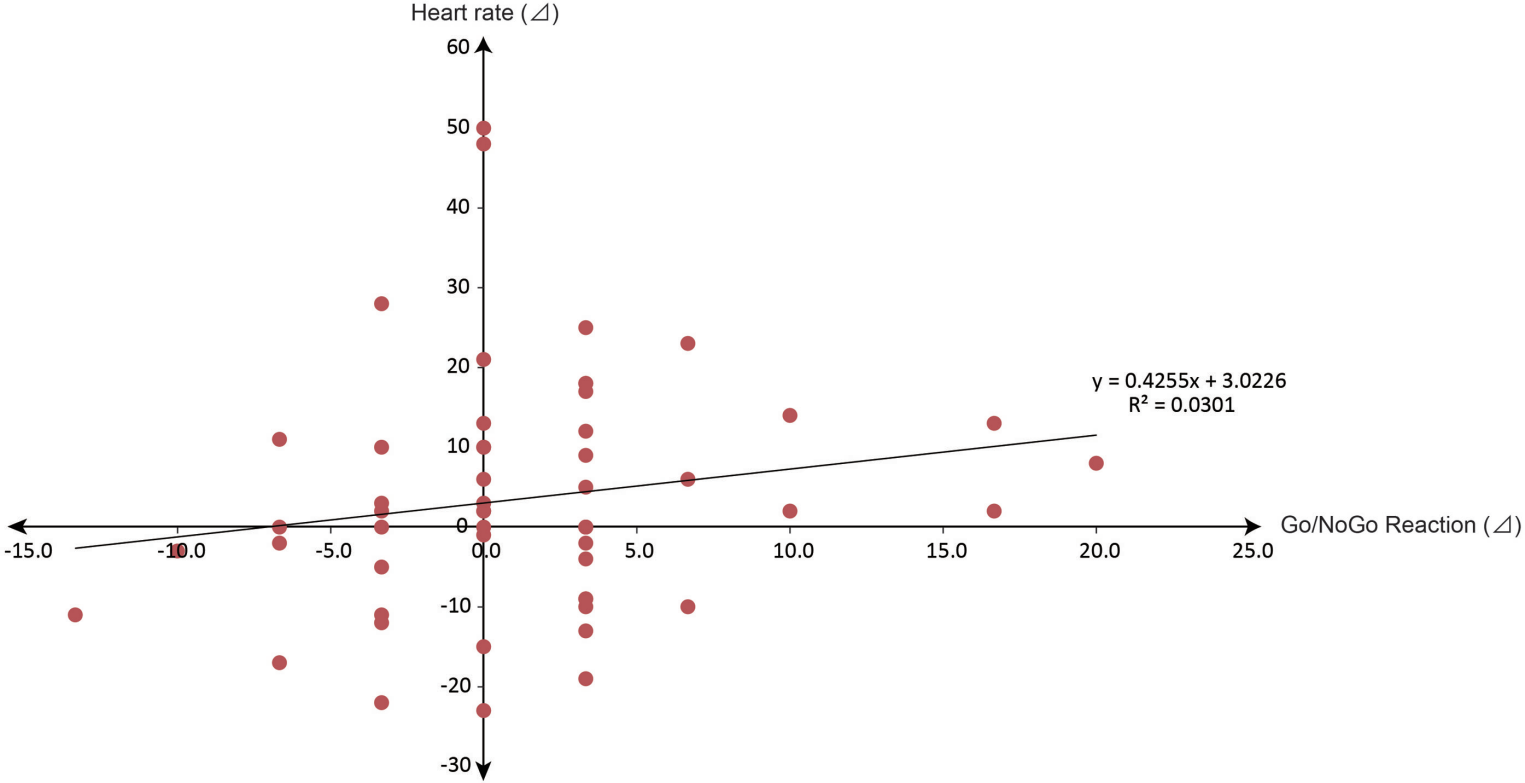
**Spearman’s rank correlation coefficient between the changes in Go/No-go reactions and those in heart rates (*R*^2^) was calculated: *R*^2^ = 0.0301, which was not significant (*r* = 0.174)**.

**Figure 5:**
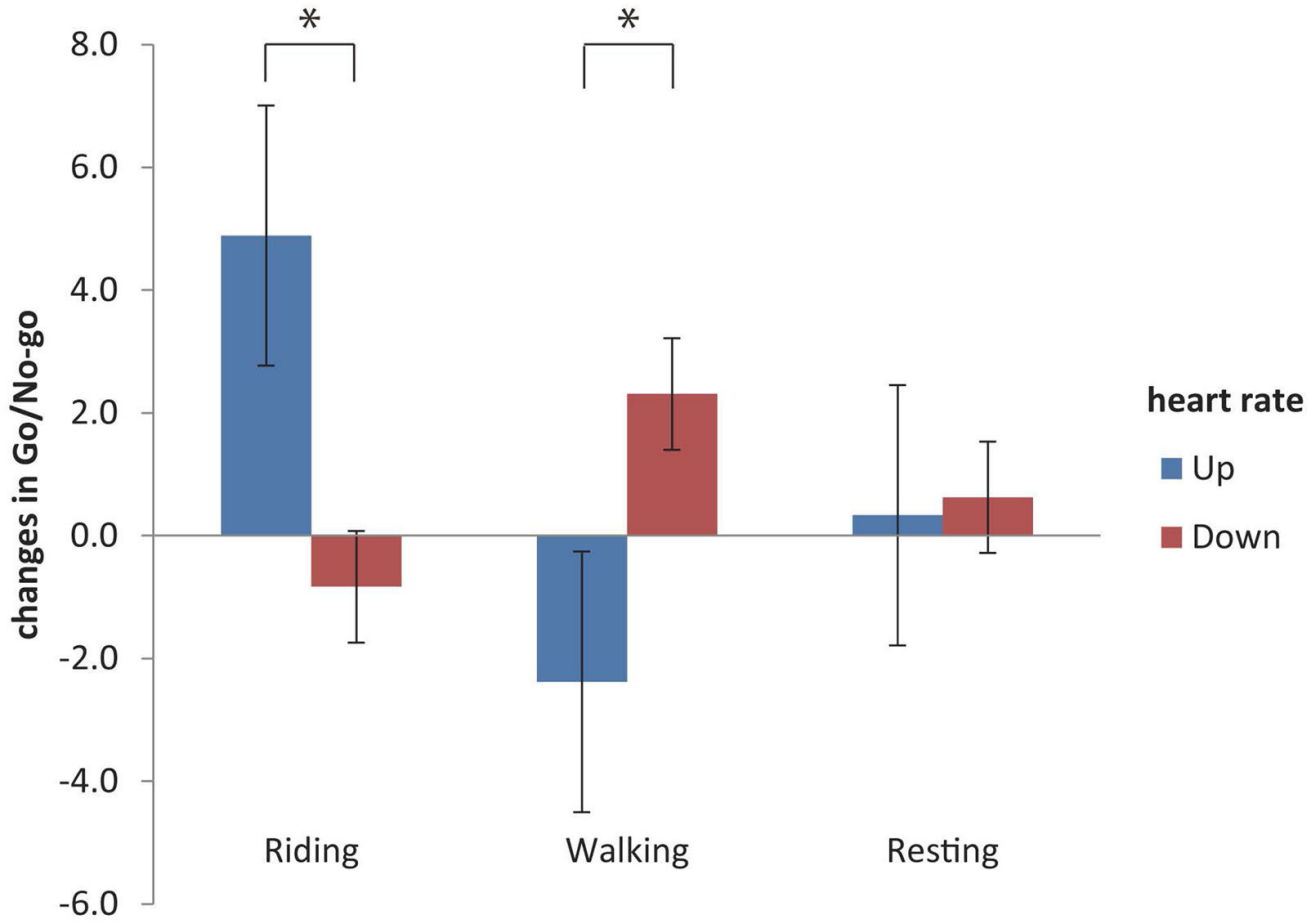
**Relation of the Go/No-go reactions with changes in the heart rates**. The changes in heart rates were calculated, and two groups were separated: the “Up” group had increases in their heart rates, while the “Down” group had decreases in their heart rates (see also Figures 1 and 5). Riding, but not walking or resting, affected the Go/No-go reactions significantly (*P* < 0.05, Welch’s test).

The changes in HRs measured by the HR monitor and transmitter were calculated as shown in delta (⊿). In total, 26 children had increased and 19 children had decreased HRs. Spearman’s rank correlation coefficient between the changes in Go/No-go reactions and those in HRs (*R*^2^) was 0.0301, which was not significant (Figure 4). However, when the increasing and decreasing HR groups (Figures 1 and 5) were compared, the riding affected the Go/No-go reactions significantly, but not the arithmetic problems.

### HRVs in Children

Heart rate variability was analyzed by fast Fourier transformation using text files from Polar ProTrainer (version 5, Polar Electro Oy, Kempele, Finland) imported into Kubios (version 2.0, 2008, Biosignal Analysis and Medical Imaging Group, University of Kuopio, Finland, MATLAB). We determined the HF component (0.15–0.4 Hz, HF), which reflects parasympathetic nervous activity, and we also determined the ratio of the LF component (0.04–0.15 Hz, LF) to the HF component (LF/HF) as an appropriate indicator of sympathetic nervous activity.

A significant increase in the HF (normalized unit) of HRV [HRV (1-1)] occurred in riders on horse B, while significant decreases in the HFs of riders were observed when riding horses A (EX. 1) and C (EX. 2) (Table 1). There were no changes in this parameter during resting or walking.

**Table 1:** **Autonomic nervous activities before and during sessions: resting, walking, and riding**.

Treatment		*n*	Pre-value: HRV (1-0)	EX. 1 [HRV (1-1)]		Pre-value: HRV (2-0)	EX. 2 [HRV (2-1)]	
**Parasympathetic component [high-frequency (normalized unit)]**								
Resting		13	43.5 ± 4.5	42.6 ± 4.3		44.9 ± 4.4	43.1 ± 5.6	
Walking		13	42.9 ± 5.1	48.3 ± 4.1		47.6 ± 4.5	46.9 ± 3.7	
Riding	A	9	42.7 ± 4.4	29.5 ± 4.3	*	44.2 ± 4.7	31.9 ± 5.2	
	B	9	43.5 ± 4.8	60.2 ± 5.7	^#^	50.7 ± 4.2	55.9 ± 3.8	
	C	9	39.9 ± 4.5	31.3 ± 4.7		40.4 ± 4.9	25.5 ± 4.1	*
**Sympathetic component (low-frequency/high-frequency)**								
Resting		13	1.60 ± 0.33	1.71 ± 0.26		1.82 ± 0.42	1.62 ± 0.33	
Walking		13	1.92 ± 0.36	4.22 ± 0.51		1.45 ± 0.42	1.74 ± 0.37	
Riding	A	9	1.93 ± 0.45	3.22 ± 0.55	*P* < 0.09	1.70 ± 0.39	3.27 ± 0.85	^#^
	B	9	1.49 ± 0.53	1.19 ± 0.18		2.23 ± 0.38	1.17 ± 0.27	*P* < 0.058
	C	9	1.58 ± 0.31	3.03 ± 0.47	^#^	1.64 ± 0.32	3.02 ± 0.81	

*Statistical analyses of the HRV data were carried out using paired t-tests, comparing values obtained during riding, walking, or resting with the pre-values. See also Figure 1*.

*Decreased significantly compared with the pre-value [HRV (1-0)] (P < 0.05).

*^#^Increased significantly compared with the pre-value [HRV (1-0)] (P < 0.05)*.

The LF/HF ratios increased significantly when riding horse C in EX. 1 and horse A in EX. 2, while riding horse B, walking or resting did not affect the LF/HF ratios significantly (Table 1).

### Accelerations

**Figure 6:**
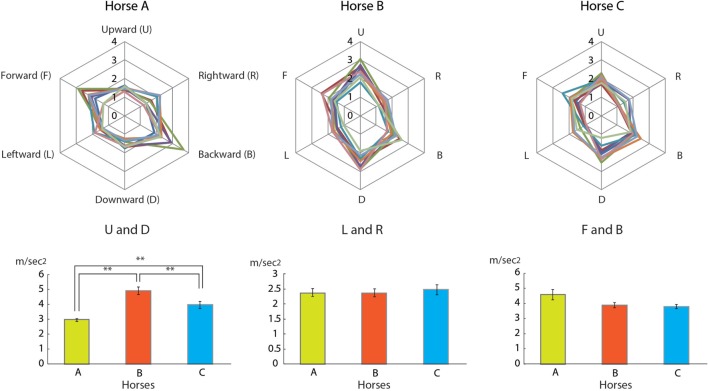
**(Upper row) Diagrams showing the three-dimensional accelerations (m/s^2^) of the three horses (A, B, and C)**. The representative number of each horse was nine. (Lower row) The significance of differences among the three horses are displayed. A significant difference (**P < 0.05, Welch’s test).

Three-dimensional accelerations, which had a range of ±2 × *g* (*g* = 9.8 m/s^2^), were measured for the horses. The three-dimensional sensors were attached to the backsides of the children. Measurements were initiated after the calibration of the sensors. A sampling rate of 50 ms was used for recording the accelerations. We collected 3,600 data points over 3 min in each riding experiment and then extracted 1,024 consecutive data points (every 51.2 s). The three-dimensional data were the averages of the maximum values of the acceleration in the *X*- (forward and backward), *Y*- (left and right), and *Z*- (up and down) axes. The calculated data for horses A, B, and C with riders during walking (upper row) and the statistical data of the acceleration on the *Z-*axis (lower row) are shown in Figure 6.

There were statistically significant differences on the *Z*-axis among horses A, B, and C (Figure 6). The *Z*-axis represents the up and down “shaking” movement, and thus riding horse B gave the children a greater up and down movement than the other horses. In contrast, the other axes of acceleration were not altered by horse walking.

## Discussion

We found that horseback riding affects some aspects of children’s behavior by investigating their abilities to perform Go/No-go tasks and solve arithmetic problems. Go/No-go tasks are a behavioral test to determine the control capacity depending on the situation, which investigates the ability to perform an appropriate action depending on the situation (Go reaction) and to demonstrate self-control appropriately (No-go reaction). Two of three horses seemed to provide an advantage to, or improve the cognitive growth of, the children in some way. Additionally, the differences in the effects of riding on children revealed that the responses of children who rode horses A and C were somewhat higher than those that rode horse B, although this is a limited number of horses.

Our results suggest that these effects are derived from vibrations produced by the horse’s motion. The motions created by a horse’s walk, trot, or canter are recognized as three-dimensional accelerations. In this study, some of the differences among the rider’s performances after riding one of the three horses might be due to these accelerations, especially those of the *Z*-axis (Figure 6, lower row). Horse B stimulated the parasympathetic nervous activity (Table 1), but not the sympathetic nervous activity. Pet ownership and dog-walking have also been shown to improve autonomic responses, such as diminished sympathetic responses to stress (20, 21). However, the performances of children who rode horse B were lower than those of children who rode on horse A or C (Figures 2 and 3), which increased sympathetic activity (Table 1), supporting previous findings of sympathetic reactivity in children (22, 23).

Horses have several innate gaits, such as the walk, trot, canter, and gallop, with each producing particular vibrations. Although we only examined the specific vibrations associated with horse walking, there were great differences in the down and up forces among the horses. The total (up and down) forces of horse B were significantly greater (Figure 6, lower row) than those of the two other horses. Nonetheless, the parasympathetic activity stimulated by riding horse B was significantly higher compared with riding the other horses. Horse B is a Kiso horse, the Japanese traditional horse whose breed originates from the Kiso Valley and the Kiso Sanmyaku Mountains, in the Nagano Prefecture of Japan. The healing effects of riding Kiso horses are legendary in Japan.

In a previous study, we conducted three-dimensional analyses of the gaits associated with horseback riding (16), describing the mechanisms underlying the effects of horseback riding on human physiology. Because the accelerations produced by horseback riding and human walking were comparable both quantitatively and qualitatively (16), horseback riding at a walking gait might generate motor and sensory inputs in humans similar to those produced by walking. In this study, however, just walking did not affect the performance of Go/No-go tasks, the ability to solve arithmetic problems, or autonomic nervous activities.

Increases in HR were associated with the improved performance of Go/No-go tasks, but not arithmetic problems. Horseback riding might have a great impact on the appropriate behavior. The arithmetic problems consisted of 30 problems of 1-digit + 1-digit addition, which should be easy for children to solve. The children performed the Go/No-go tasks and the arithmetic problems twice, pre- and post-activity (Figure 1). In fact, there was no significant difference in the solving of arithmetic problems between the pre- and the post-tasks (Figure 3A). It was possible that children learned the tests and performed better on them. Thus, the effects of riding were compared with those of walking and resting (controls). The Go/No-go tasks might be harder than the arithmetic problems and thus cause a more extensive activation of the sympathetic nervous system.

Many reports have demonstrated the benefits of horseback riding with respect to enhancing physical health (7), as well as the mental effects (10, 12). There are also many possible effects of human–animal interactions, such as horseback riding, on child development (24). For instance, the ability to make considered decisions or come to sensible conclusions, which we described in this study, and the ability to appreciate and respond to complex emotional influences and non-verbal communication, which requires further research to be understood.

## Conclusion

The important benefits of horseback riding for children and human health appear to be caused by the horse’s vibrations, which may be different among horses. Riding particular horses or breeds might improve the ability to recognize the appropriate action depending on the situation (Go reaction) and the appropriately self-control (No-go reaction) in children, possibly through the activation of the sympathetic nervous system. Some horse riding may reduce stress through the activity of the parasympathetic nervous system.

## Author Contributions

NO, KK, KK, KM, MF, JA, HU, and MO contributed to the conception and design of the work. NO and MO were responsible for data acquisition. NO, KK, KM, MF, and MO interpreted the data and HU and JA did the analyses. NO, KK, KM, MF, and MO drafted and revised the manuscript which was then finalized by NO and MO.

## Conflict of Interest Statement

The authors declare that the research was conducted in the absence of any commercial or financial relationships that could be construed as a potential conflict of interest.
